# Association between hydroxocobalamin administration and acute kidney injury after smoke inhalation: a multicenter retrospective study

**DOI:** 10.1186/s13054-019-2706-0

**Published:** 2019-12-23

**Authors:** François Dépret, Clément Hoffmann, Laura Daoud, Camille Thieffry, Laure Monplaisir, Jules Creveaux, Djillali Annane, Erika Parmentier, Daniel Mathieu, Sandrine Wiramus, Dominique Demeure DIt Latte, Aubin Kpodji, Julien Textoris, Florian Robin, Kada Klouche, Emmanuel Pontis, Guillaume Schnell, François Barbier, Jean-Michel Constantin, Thomas Clavier, Damien du Cheyron, Nicolas Terzi, Bertrand Sauneuf, Emmanuel Guerot, Thomas Lafon, Alexandre Herbland, Bruno Megarbane, Thomas Leclerc, Vincent Mallet, Romain Pirracchio, Matthieu Legrand

**Affiliations:** 1 Department of Anesthesiology and Critical Care and Burn Unit, AP-HP, GH Saint Louis-Lariboisière, Paris, France; 2 UMR INSERM 942, Institut National de la Santé et de la Recherche Médicale (INSERM), Paris, France; 3 F-CRIN, INICRCT network, Paris, France; 4 Paris Diderot University, F-75475 Paris, France; 5 Burn Center, Percy Military Teaching Hospital, BP 406, 101, avenue Henri-Barbusse, 92141 Clamart CEDEX, France; 6 Intensive Care Unit and Hyperbaric Center, Lille University Hospital, F-59037 Lille CEDEX, France; 7 General ICU, Service de Réanimation, Hôpital Raymond Poincaré, Laboratory of Infection and Inflammation, U1173, AP-HP, University of Versailles SQY and INSERM, 104 Boulevard Raymond Poincaré, 92380 Garches, France; 8 Centre de traitement des grands brûlés Hopital de la Conception APHM, 147 boulevard Baille, 13005 Marseille, France; 9 Intensive Care Unit, Anaesthesia and Critical Care Department, Hôtel Dieu—HME, CHU Nantes, Nantes, France; 10 Centre de traitement des grands brûlés Hopital de Mercy,1 Allée du Château, 57245 Ars-Laquenexy—C.H.R Metz-, Thionville, France; 11 Anesthesiology and Critical Care Medicine, Hospices Civils de Lyon—Université Claude Bernard Lyon 1, Lyon, France; 12 Anesthesiology and Critical Care Medicine, CHU Bordeaux, Place Amélie Raba Léon, 33000 Bordeaux, France; 13 Intensive Care Medicine Department, University of Montpellier Lapeyronie Hospital, 371, Av Doyen Gaston Giraud, 34295 Montpellier, France; 14 Intensive Care Medicine Department, CHU de Rennes, 2 rue Henri Le Guilloux, 35033 Rennes CEDEX 9, France; 15 Service de réanimation médico-chirurgicale, Groupe Hospitalier du Havre—Hôpital Jacques Monod, Montivilliers, France; 16 Medical Intensive Care Unit, La Source Hospital, CHR Orléans, Orléans, France; 17 Department of Perioperative Medicine, University Hospital of Clermont-Ferrand, Clermont-Ferrand, France; 18 Department of Anesthesiology and Critical Care, Rouen University Hospital, Rouen, France; 19 Normandie Univ, UNIROUEN, INSERM U1096, Rouen, France; 20 Medical Intensive Care Unit, Caen University Hospital, Avenue côte de Nacre, 14033 Caen CEDEX, France; 21 Service de Réanimation Médicale, Centres Hospitaliers Universitaires Grenoble Alpes, Grenoble, France; 22 Service de Réanimation Médicale Polyvalente, Centre Hospitalier Public du Cotentin, BP 208, 50102 Cherbourg-Octeville, France; 23 Service de Réanimation Médicale, Hôpital Européen Georges-Pompidou, Assistance Publique-Hôpitaux de Paris, Paris, France; 24 Faculté de Médecine, Université Paris Descartes, Paris, France; 25 Département des urgences, service des urgences, SAMU, CHU de Limoges, 87042 Limoges CEDEX, France; 26 Inserm CIC 1435, 87042 Limoges, France; 27 Intensive Care Unit, Saint Louis Hospital, La Rochelle, France; 28 Service de réanimation médicale et toxicologie, Hôpital Lariboisière, Assistance Publique-Hôpitaux de Paris, Paris, France; 29 Service d’hépato gastro entérologie Hôpital Cochin, hépato Cochin, Assistance Publique-Hôpitaux de Paris, Paris, France; 30 Department of Anesthesia and perioperative care, University of California San Francisco, San Francisco, USA; 31 Department of Anesthesiology and Perioperative care Parnassus hospital, UCSF, San Francisco, USA

**Keywords:** Smoke inhalation, Acute kidney injury, Intensive care unit, Mortality, Burn, Hydroxocobalamin

## Abstract

**Background:**

The use of hydroxocobalamin has long been advocated for treating suspected cyanide poisoning after smoke inhalation. Intravenous hydroxocobalamin has however been shown to cause oxalate nephropathy in a single-center study. The impact of hydroxocobalamin on the risk of acute kidney injury (AKI) and survival after smoke inhalation in a multicenter setting remains unexplored.

**Methods:**

We conducted a multicenter retrospective study in 21 intensive care units (ICUs) in France. We included patients admitted to an ICU for smoke inhalation between January 2011 and December 2017. We excluded patients discharged at home alive within 24 h of admission. We assessed the risk of AKI (primary endpoint), severe AKI, major adverse kidney (MAKE) events, and survival (secondary endpoints) after administration of hydroxocobalamin using logistic regression models.

**Results:**

Among 854 patients screened, 739 patients were included. Three hundred six and 386 (55.2%) patients received hydroxocobalamin. Mortality in ICU was 32.9% (*n* = 243). Two hundred eighty-eight (39%) patients developed AKI, including 186 (25.2%) who developed severe AKI during the first week. Patients who received hydroxocobalamin were more severe and had higher mortality (38.1% vs 27.2%, *p* = 0.0022). The adjusted odds ratio (95% confidence interval) of AKI after intravenous hydroxocobalamin was 1.597 (1.055, 2.419) and 1.772 (1.137, 2.762) for severe AKI; intravenous hydroxocobalamin was not associated with survival or MAKE with an adjusted odds ratio (95% confidence interval) of 1.114 (0.691, 1.797) and 0.784 (0.456, 1.349) respectively.

**Conclusion:**

Hydroxocobalamin was associated with an increased risk of AKI and severe AKI but was not associated with survival after smoke inhalation.

**Trial registration:**

ClinicalTrials.gov, NCT03558646

## Introduction

Patients with smoke inhalation are at a high risk of death or major morbidities. Cyanide is a documented cause for rapid death after intoxication from smoke [1]. Several cyanide antidotes have been proposed (i.e., sodium nitrite, amyl nitrite, sodium thiosulfate, 4-dimethylaminophenol, dicobalt edetate, and hydroxocobalamin) with utilization varying across countries [2]. Several experts suggested using hydroxocobalamin as the first-line treatment after suspected cyanide intoxication due to its perceived good safety profile [3]. However, other experts raised concerns about liberalizing the use of hydroxocobalamin after smoke inhalation due to a lack of data on both efficacy and tolerance. They have called for additional investigations [4, 5].

Recently, nephrotoxicity of hydroxocobalamin due to oxalate nephropathy was reported in a single-center study among burn patients [4]. The objective of this cohort study was to assess the association between the use of hydroxocobalamin and the risk of acute kidney injury (AKI) and death in intensive care unit (ICU) patients with smoke inhalation.

## Methods

### Study population and settings

We conducted a retrospective, multicenter study in 21 ICUs in France. All consecutive adult (≥ 18 years old) patients admitted between January 2011 and December 2017 with the final diagnosis of smoke inhalation were included in this study. Patients discharged alive at home within 24 h from admission (reflecting absence of severity) were excluded. Patient files were retrieved using our national coding for smoke inhalation injury (code CIM T599 for all patients and associated: X00·0, X 09·0, X09·9, X47·0, X47·08, X47·9, X67·0, X67·9, T58) (Additional file 1: Table S1). This academic investigator-driven study was registered at ClinicalTrials.gov, NCT03558646. The study protocol was approved by our local research ethical committee (Comité de Protection des Personnes 2013/17NICB). The use of patient’s data was allowed in cases of death and/or when proxies could not be contacted.

### Primary outcome

The primary outcome was AKI. AKI was defined according to Kidney Disease Improving Global Outcome (KDIGO) within 7 days following admission using the serum creatinine criteria [6].

### Secondary outcome

Secondary outcomes were severe AKI (severe AKI defined patients by AKI stage 2 or 3), major associated kidney events (MAKE) in the ICU, which includes death and/or renal replacement therapy (RRT), and/or persistent AKI at ICU discharge. Persistent AKI was defined as an elevated SCreat level from baseline by > 1.5-fold or > 0.3 mg/dL (26.3 μmol/L) at ICU discharge or RRT at ICU discharge and survival in the ICU.

### Data collections and definitions

Data were collected through a standardized case report form. Each form was manually encoded in each participating center using the initials and year of birth of the patient. Admission serum creatinine (SCreat) was used for baseline SCreat. Severe burn was defined as total body surface area (TBSA) burn ≥ 20% and/or deep TBSA burn ≥ 10% with organ support on admission (i.e., mechanical ventilation or need for vasopressors) [7]. When a fiber-optic bronchoscopy was performed, inhalation injury was classified according to fiber-optic bronchoscopy inhalation injury classification [8].

### Sample size and statistical analysis

Due to the exploratory design of this study, no sample size could be calculated. Categorical variables were presented using percentages and counts, and continuous variables were presented using means and standard deviations or medians with the 25th and 75th percentiles. Categorical variables were compared using the chi-square test or Fisher’s exact test as appropriate. Continuous variables were compared using the Student *t* test or the Mann–Whitney *U* test as appropriate.

Actuarial mortality was plotted using the Kaplan–Meier estimator. Administrative censoring was applied at day 90. The impact of hydroxocobalamin administration on AKI was estimated using a multivariate logistic regression model adjusting age, comorbidities, aminoglycoside, vancomycin and iodine contrast agent utilization during hospitalization, peak value of creatinine phosphokinase, prehospital cardiac arrest, prehospital minimal GCS, severe burn, initial sequential organ failure assessment (SOFA) score without the renal item, lactate at admission, need for catecholamine infusion at admission, the SAPS2 score calculated over the first 24 h, the interaction between hydroxocobalamin administration and prehospital cardiac arrest, and the center. The impact of hydroxocobalamin administration on secondary outcomes (severe AKI, MAKE, survival) was estimated using a multivariate logistic regression model adjusting for the same confounders. An interaction term between prehospital cardiac arrest and hydroxocobalamin was also introduced in the model. Odds ratios were provided together with their 95% confidence intervals.

Missing data were handled using multivariate imputation by chained equations (mice package for R, 50 imputations) [9].

Because plasma lactate level was shown to correlate with cyanide, we performed subgroup analysis in patients with plasma lactate level at admission above median of the entire cohort and above 8 mmol/L [10, 11]. A subgroup analysis was also performed in patients with severe burns.

## Results

Among 854 patients with smoke inhalation, 739 were included in the analysis after exclusion of patients discharged at home within 24 h. Three hundred eighty-six (55.2%) patients received hydroxocobalamin. Patient’s characteristics and comparison between patients who received hydroxocobalamin and those who did not are presented in Table 1. The number of patients per center is summarized in Additional file 2: Table S2.

**Table 1:** Comparison between patients with or without hydroxocobalamin

Characteristics	All patients, *N* = 739	Hydroxocobalamin, *N* = 386	No hydroxocobalamin, *N* = 353	*p*
At admission				
- Age in years	50 (36–63)	50 (38–62)	48 (33–64)	0.4858
- Sex female, n (%)	271 (36.7)	140 (36.3)	131 (37.1)	0.9335
- BMI in kg/m^2^	25 (22–28)	24 (22–28)	25 (22–28)	0.2416
- Prehospital cardiac arrest (%)	46 (6.2)	42 (10.9)	4 (1.1)	< 0.0001
- Prehospital GSC /15	15 (9–15)	13 (5–15)	15 (14–15)	< 0.0001
Comorbidities				
- CKD, *n* (%)	6 (0.8)	6 (1.6)	0 (0)	0.0315
- CHT, *n* (%)	141 (19.1)	71 (18.4)	70 (19.9)	0.6872
- Diabetes mellitus, *n* (%)	54 (7.3)	33 (8.5)	21 (6)	0.2243
- Peripheral artery disease, *n* (%)	22 (3)	9 (2.3)	13 (3.7)	0.3882
- CHF, *n* (%)	33 (4.5)	20 (5.2)	13 (3.7)	0.4197
Burn characteristic				
- Burn, *n* (%)	577 (78.1)	286 (74.1)	291 (82.4)	0.0081
- TBSA %	20 (3–47)	15 (0–45)	24 (6–50)	0.0163
- Deep burn TBSA %	9 (0–30)	5 (0–30)	10 (0–32)	0.1985
SOFA at admission	4 (1–7)	5 (2–8)	2 (0–5)	< 0.0001
MAP in mmHg	86 (72–101)	86 (68–101)	86 (73–101)	0.4383
Vasopressors, *n* (%)	226 (30.6)	153 (39.6)	73 (20.7)	< 0.0001
HbCO %	3.6 (1.9–9.7)	7 (3–15)	3 (2–5)	< 0.0001
Biological data				
- Plasma lactate in mmol/L	3.0 (1.8–5.2)	3.5 (2.1–6)	2.6 (1.4–4.1)	< 0.0001
- Serum creatinine at admission in μmol/L	76 (59–101)	82 (63–106)	71 (56–93)	0.0031
- Maximal serum creatinine in μmol/L	100 (73–162)	108 (77–182)	90 (71–137)	0.0027
Inhalation fibroscopic status, *n* (%)	305 (41.3)	105 (27.5)	200 (56.7)	< 0.0001
- Grade 0, *n*	1 (0.1)	0 (0)	1 (0.3)	1
- Grade 1, *n*	121 (16.4)	31 (8)	90 (25.5)	< 0.0001
- Grade 2, *n*	110 (14.9)	37 (9.6)	73 (20.7)	< 0.0001
- Grade 3, *n*	73 (9.9)	37 (9.6)	36 (10.2)	0.8764
During ICU hospitalization				
- In-ICU mortality, *n* (%)	243 (32.9)	147 (38.1)	96 (27.2)	0.0022
- AKI in the first week, *n* (%)	288 (39)	166 (43)	122 (34.6)	0.0229
- Stage of AKI				
- Stage 1, *n* (%)	102 (13.8)	52 (13.5)	50 (14.2)	0.8682
- Stage 2, *n* (%)	39 (5.3)	22 (5.7)	17 (4.8)	0.7099
- Stage 3, *n* (%)	147 (19.9)	92 (23.8)	55 (15.6)	0.0066
- Severe AKI, *n* (%)	186 (25.2)	114 (29.5)	72 (20.4)	0.0055
RRT at day 7, *n* (%)	136 (18.8)	86 (22.3)	50 (14.2)	0.006
- RRT in ICU, *n* (%)	183 (24.8)	107 (27.7)	76 (21.5)	0.0626
- MAKE, *n* (%)	313 (42.4)	187 (48.4)	126 (35.7)	0.0006
- Shock in ICU, *n* (%)	402 (54.4)	225 (58.3)	176 (50)	0.0261
- Length of stay in ICU in days	15 (3–44)	11 (2–36)	22 (3–50)	0.0161
- SAPS2	42 (27–60)	49 (31–77)	37 (23–54)	< 0.0001
- In-ICU survival, *n* (%)	496 (67.1)	239 (61.9)	257 (72.8)	0.0022
Nephrotoxic in ICU				
- Aminoglycoside during hospitalization	188 (25.4)	81 (21)	107 (30.3)	0.0047
- Glycopeptide during hospitalization	41 (5.5)	16 (4.1)	25 (7.1)	0.1138
- Contrast agent	74 (10)	48 (12.4)	26 (7.4)	0.0299

All data are expressed as median ± 25–75 interquartile or percentage (%)

*BMI* body mass index, *GCS* Glasgow coma scale, *CKD* chronic kidney disease, *CHT* chronic hypertension, *CHF* chronic heart failure, *TBSA* total body surface area, *SOFA* sequential organ failure assessment, *MAP* mean arterial pressure, *HbCO* carboxy hemoglobin, *ICU* intensive care unit, *AKI* acute kidney injury, *Severe AKI* AKI stage 2 and 3, *RRT* renal replacement therapy, *MAKE* major associated kidney events, *Shock in ICU* catecholamine need during ICU stay, *SAPS2* simplified acute physiology score 2

### Acute kidney injury

**Fig. 1:**
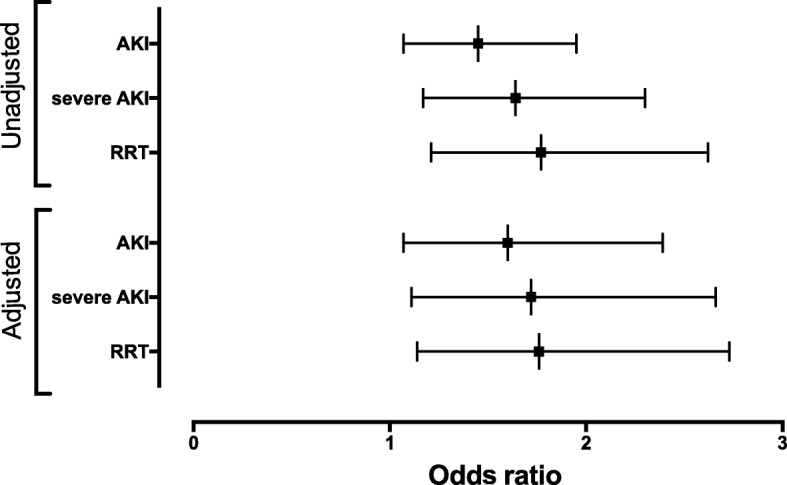
Non-adjusted and adjusted odds ratio (lower confidence interval, upper confidence interval) of hydroxocobalamin for AKI (upper panel) and severe AKI (lower panel). Adjusted on the following variables: age, peripheral arterial obstructive disease, diabetes mellitus, chronic hypertension, chronic kidney disease, prehospital cardiac arrest, severe burn, SOFA score without kidney item, vasopressors at admission, prehospital Glasgow coma scale, plasmatic lactate level at admission, maximum creatinine phosphokinase plasmatic level, contrast agent, use of vancomycin and aminoglycosides, and simplified acute physiology score 2

Two hundred eighty-eight (39%) patients developed AKI in the first week, including 186 (25.2%) patients with severe AKI. In univariate analysis, the use of hydroxocobalamin was associated with AKI, severe AKI, and RRT (Table 1). After adjusting for potential confounders, hydroxocobalamin remained associated with AKI and severe AKI and RRT (Table 2, Fig. 1 and Additional file 3: Table S3). Severe burns, aminoglycoside administration, admission plasma lactate level, chronic hypertension, and SAPS2 were also associated with AKI (Table 2).

**Table 2:** Multivariate analyses of factors associated with AKI and severe AKI

Variable	Adjusted odds ratio	LCI	UCI	*p*
AKI				
Hydroxocobalamin	1.597	1.055	2.419	0.027
Severe burn	2.91	1.841	4.601	< 0.001
SOFA score without renal item	1.003	0.891	1.129	0.963
Prehospital GCS	1.064	0.997	1.136	0.063
Aminoglycoside during hospitalization	2.903	1.184	4.473	< 0.001
Glycopeptide during hospitalization	1.731	0.773	3.877	0.182
Admission plasma lactate	1.105	1.034	1.182	0.003
Age	1.009	0.997	1.021	0.154
Peripheral arterial obstructive disease	0.913	0.308	2.713	0.87
Diabetes mellitus	1.487	0.726	3.046	0.278
Chronic hypertension	2.134	1.248	3.649	0.006
CKD	0.947	0.119	7.504	0.959
Prehospital cardiac arrest	0.418	0.025	7.096	0.546
Vasopressor at admission	1.778	0.93	3.396	0.081
Maximum CPK	1.081	0.997	1.242	0.269
Contrast agent	1.445	0.803	2.601	0.219
SAPS2	1.021	1.006	1.036	0.009
Center	1.014	0.976	1.053	0.484
Interaction between prehospital cardiac arrest and hydroxocobalamin	2.245	0.125	40.367	0.583
Severe AKI				
Hydroxocobalamin	1.772	1.137	2.762	0.012
Age	1.006	0.993	1.019	0.394
Peripheral arterial obstructive disease	0.552	0.183	1.668	0.292
Diabetes mellitus	1.201	0.582	2.479	0.619
Chronic hypertension	2.045	1.168	3.579	0.012
Prehospital cardiac arrest	0.775	0.043	14.12	0.863
Severe burn	2.157	1.276	3.645	0.004
SOFA score at admission without renal item	1.047	0.924	1.185	0.471
CKD	2.407	0.285	20.299	0.418
Vasopressor at admission	1.298	0.653	2.579	0.456
Prehospital GCS	1.04	0.973	1.11	0.248
Admission plasma lactate	1.151	1.062	1.248	0.001
Maximum CPK	1.099	0.904	1.337	0.324
Aminoglycoside during hospitalization	2.418	1.539	3.801	< 0.001
Contrast agent	0.889	0.476	1.663	0.713
Glycopeptide during hospitalization	2.873	1.318	6.261	0.008
SAPS2	1.02	1.006	1.035	0.006
Center	1.042	0.998	1.089	0.061
Interaction between prehospital cardiac arrest and hydroxocobalamin	0.412	0.02	8.331	0.563

*AKI* acute kidney injury, *LCI* lower confidence interval, *UCI* upper confidence interval, *p p* value, *SOFA score* sequential organ failure assessment, *GCS* Glasgow coma scale, *CKD* chronic kidney disease, *CPK* creatinine phosphokinase, *SAPS2* simplified acute physiology score 2

Three hundred and thirteen (42.4%) patients met the criteria for MAKE during ICU stay. Hydroxocobalamin was not associated with the risk of MAKE in multivariate analysis (OR = 0.784, LCI = 0.456, UCI = 1.349, *p* = 0.379). Factors associated with MAKE in ICU are summarized in Additional file 4: Table S4.

### ICU mortality

**Fig. 2:**
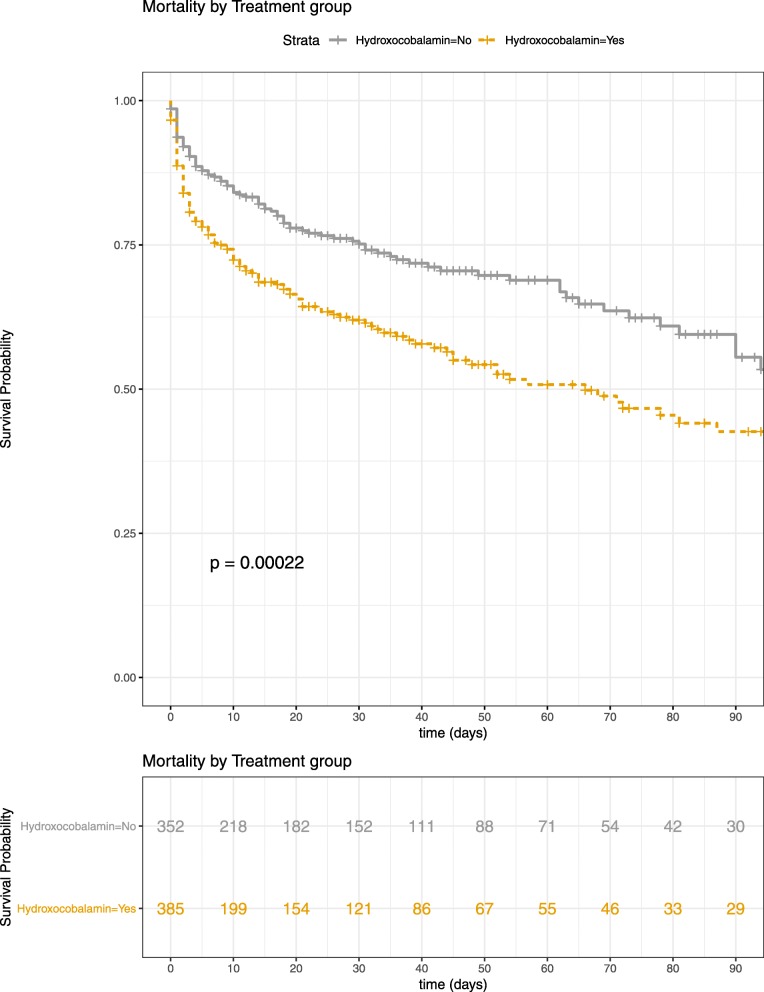
Unadjusted Kaplan–Meier curve of survival incidence during the 90 days following admission between patients who received hydroxocobalamin and those who did not

Mortality in ICU was 32.9% (*N* = 243). Patients who received hydroxocobalamin had higher severity scores and higher mortality rate. Factors associated with mortality in univariate analysis are summarized in Table 3 and Fig. 2. In multivariate analysis, hydroxocobalamin was not associated with survival (OR = 1.114, LCI = 0.691, UCI = 1.797, *p* = 0.657). Age, severe burns, SAPS2, plasma lactate level at admission, and the center were associated with mortality in ICU (Table 4). Three hundred and ninety-two patients (53%) had admission plasma lactate level above the median (median = 3.0 [1.8–5.2] mmol/L), and 74 patients had admission plasma lactate level ≥ 8 mmol/L (median = 10.5 [8.9–12.8] mmol/L) (among 681 (92.2%) patients with plasma lactate available at admission, Additional file 5: Table S5). No association between hydroxocobalamin use and survival was observed after adjustment for confounding factors in the subgroups of patients with admission plasma lactate level above the median (OR = 0.76; LCI = 0.398, UCI = 1.451, *p* = 0.403) or in the group of patients with admission plasma lactate level ≥ 8 mmol/L (OR = 2.604, LCI = 0.361, UCI = 18.79, *p* = 0.336). In multivariate analysis, hydroxocobalamin was neither associated with survival (OR = 0.85, LCI = 0.5, UCI = 1.447, *p* = 0.549) among 405 (54.8%) patients with severe burns (Additional file 6: Table S6).

**Table 3:** Patient characteristics

Characteristics	All patients, *N* = 739	Survivors, *N* = 496	Non-survivors, *N* = 243	*p*
At admission				
- Age in years	50 (36–63)	46 (33–59)	56 (43–72)	< 0.0001
- Sex female, *n* (%)	271 (36.7)	180 (36.3)	91 (37.4)	0.8214
- BMI in kg/m^2^	25 (22–28)	24 (22–28)	25 (22–29)	0.0822
- Prehospital cardiac arrest (%)	46 (6.2)	11 (2.2)	35 (14.4)	< 0.0001
- Prehospital GSC /15	15 (9–15)	15 (13–15)	14 (3–15)	< 0.0001
Comorbidities				
- CKD, *n* (%)	6 (0.8)	2 (0.4)	4 (1.6)	0.0948
- Hypertension, *n* (%)	141 (19.1)	81 (16.3)	60 (24.7)	0.0088
- Diabetes mellitus, *n* (%)	54 (7.3)	28 (5.6)	26 (10.7)	0.0198
- CAOD, *n* (%)	22 (3)	7 (1.4)	15 (6.2)	0.0008
- CHF, *n* (%)	33 (4.5)	20 (4)	13 (5.3)	0.4858
Burn characteristic				
- Burn, *n* (%)	577 (78.1)	357 (72)	220 (90.5)	< 0.0001
- TBSA %	20 (3–47)	12 (0–30)	50 (20–73)	< 0.0001
- 3rd degree TBSA %	9 (0–30)	2 (0–15)	33 (10–58)	< 0.0001
SOFA at admission	4 (1–7)	2 (1–5)	7 (3–10)	< 0.0001
MAP in mmHg	86 (72–101)	89 (76–102)	80 (62–97)	< 0.0001
Vasopressor, *n* (%)	226 (30.6)	90 (18.1)	136 (56)	< 0.0001
Hydroxocobalamin, *n* (%)	386 (52.2)	239 (48.2)	147 (60.5)	0.0021
HbCO %	4 (2–10)	4 (2–11)	3 (2–7)	0.0323
Biological data				
- Plasmatic lactate in mmol/L	3.0 (1.8–5.2)	2.5 (1.4–3.8)	4.8 (3–7.6)	< 0.0001
- Serum creatinine in μmol/L	76 (59–101)	71 (56–88)	100 (72–123)	< 0.0001
- Maximal serum creatinine in μmol/L	100 (73–162)	84 (68–113)	137 (102–212)	< 0.0001
Inhalation fibroscopic status (%)	305 (41.3)	175 (35.3)	130 (53.5)	< 0.0001
- Grade 0, *n*	1 (0.1)	1 (0.2)	0 (0)	1
- Grade 1, *n*	121 (16.4)	90 (18.2)	31 (12.8)	0.0795
- Grade 2, *n*	110 (14.9)	67 (13.5)	43 (17.7)	0.1638
- Grade 3, *n*	73 (9.9)	17 (3.4)	56 (23.1)	< 0.0001
- During ICU hospitalization				
- AKI in the first week, *n* (%)	288 (39)	126 (25.4)	162 (66.7)	< 0.0001
- Stage of AKI day 7				
- Stage 1, *n* (%)	102 (13.8)	63 (12.7)	39 (16)	0.2601
- Stage 2, *n* (%)	39 (5.3)	16 (3.2)	23 (9.5)	0.0007
- Stage 3, *n* (%)	147 (19.9)	47 (9.5)	100 (41.2)	< 0.0001
- Severe AKI	186 (25.2)	63 (12.7)	123 (50.6)	< 0.0001
- RRT at day 7, *n* (%)	136 (18.8)	44 (8.9)	92 (37.9)	< 0.0001
- RRT in ICU, *n* (%)	183 (24.8)	58 (11.7)	125 (51.4)	< 0.0001
- MAKE, *n* (%)	313 (42.4)	69 (13.9)	243 (100)	< 0.0001
- Shock in ICU, *n* (%)	402 (54.4)	191 (38.5)	211 (86.8)	< 0.0001
- Length of stay in ICU	15 (3–44)	25 (4–50)	6 (1–24)	0.0001
- SAPS2	42 (27–60)	32 (22–46)	61 (46–75)	< 0.0001
Nephrotoxic in ICU				
- Aminoglycoside	188 (25.4)	115 (23.2)	73 (42.9)	0.0548
- Vancomycin	41 (5.5)	22 (4.4)	19 (7.8)	0.4011
- Contrast product	74 (10)	41 (8.3)	33 (13.6)	0.0331

All data are expressed as median ± 25–75 interquartile or percentage (%)

*BMI* body mass index, *GCS* Glasgow coma scale, *CKD* chronic kidney disease, *CAOD* chronic arterial occlusive disease, *CHF* chronic heart failure, *TBSA* total body surface area, *SOFA* sequential organ failure assessment, *MAP* mean arterial pressure, *HbCO* carboxy hemoglobin, *ICU* intensive care unit, *AKI* acute kidney injury, *RRT* renal replacement therapy, *MAKE* major associated kidney events, *SAPS2* simplified acute physiology score 2

**Table 4:** Multivariate analyses of factors associated with ICU mortality

ICU mortality				
Variable	Adjusted odds ratio	LCI	UCI	*p*
Hydroxocobalamin	1.114	0.691	1.797	0.657
Age	1.037	1.023	1.05	< 0.001
Prehospital cardiac arrest	1.091	0.07	17.126	0.95
Severe burn	4.792	2.665	8.617	< 0.001
SOFA score at admission	1.074	0.942	1.225	0.283
Admission plasma lactate	1.256	1.154	1.366	< 0.001
Vasopressors at admission	1.571	0.737	3.346	0.241
Prehospital GCS	1.033	0.961	1.11	0.383
SAPS2	1.039	1.02	1.059	< 0.001
Center	1.084	1.033	1.137	0.001
Interaction between prehospital cardiac arrest and hydroxocobalamin	6.327	0.354	112.993	0.209

*ICU* intensive care unit, *LCI* lower confidence interval, *UCI* upper confidence interval, *p p* value, *SOFA score* sequential organ failure assessment score, *GCS* Glasgow coma scale, *SAPS2* simplified acute physiology score 2

## Discussion

In this retrospective, multicenter study, we observed a higher risk of AKI and severe AKI associated with administration of hydroxocobalamin after adjustment for potential confounding factors. Hydroxocobalamin was not associated with improved survival.

Cyanide poisoning has been suspected to account for deaths after smoke inhalation [12]. Hydroxocobalamin (Cyanokit®, Merck Santé SAS) is approved by the Food and Drug Administration for the treatment of cyanide poisoning. Hydroxocobalamin chelates cyanide to form cyanocobalamin, which is excreted by the kidneys. Experts have advocated the use of cyanide antidotes after smoke inhalation with suspected cyanide intoxication. While preclinical data have suggested improved hemodynamics with hydroxocobalamin in animal models of cyanide intoxication, clinical data are very limited. In two retrospective studies, Borron et al. and Fortin et al. described the outcome of patients receiving hydroxocobalamin after smoke inhalation injury. However, the absence of control groups precluded drawing any conclusion with respect to the impact of hydroxocobalamin on outcome [13, 14]. Nguyen et al. reported that the use of hydroxocobalamin was associated with fewer episodes of pneumonia while mortality was unaffected [15]. However, the association between the prevention of pneumonia lies on very low level of evidence. Other factors, especially with respect to pneumonia preventive strategies, may have accounted for the observation of lower incidence of pneumonia in this before-and-after retrospective study.

In the current study, we observed an association between hydroxocobalamin and the risk of AKI and severe AKI. These results further suggest the potential nephrotoxicity of the drug. We previously showed that hydroxocobalamin induces oxaluria with a risk of oxalate nephropathy in burn patients [4]. Kidney biopsies confirmed renal calcium oxalate crystal deposits. Oxaluria was also reported in healthy volunteers and animals receiving hydroxocobalamin [16]. Nitric oxide chelation could represent an additional mechanism of renal toxicity of hydroxocobalamin. In the absence of cyanide, hydroxocobalamin binds to nitric oxide. Nitric oxide is a key factor of the renal microcirculatory regulation, and inhibition of the nitric oxide pathway was shown to impair renal perfusion and oxygenation and cause kidney damage [17–19].

Cyanide is a rapidly lethal mitochondrial poison. The plasma cyanide level is difficult to measure and not readily available, and none of the patients included in this study had a plasma cyanide dosage. Increased serum lactate level > 8 mmol/L was reported to be a fair surrogate biomarker of cyanide poisoning as a biomarker of mitochondrial dysfunction [10]. In our study, hydroxocobalamin had no impact in the subgroup of patients with high plasma lactate level. The lack of evidence of any association between hydroxocobalamin and survival in our study and the risk of AKI question the risk-benefit ratio in these patients. Of note, hydroxocobalamin costs about €1000/$1200 per vial.

We acknowledge the limitations of our study. First, the retrospective nature introduced some bias. Performing a randomized control trial in this setting would be highly difficult due to the low incidence of smoke inhalation and the existing cognitive biases toward the potential protective effects of hydroxocobalamin. Second, the relative low number of patients with very high plasma lactate level precludes drawing firm conclusion in patients with a high probability of cyanide poisoning and in most severe patients. Third, we could not confirm cyanide levels in any patient, which makes it hard to determine if hydroxocobalamin could benefit patients with confirmed cyanide poisoning. Finally, rhabdomyolysis may be a confounding factor with the elevated serum creatinine. However, we did not find any correlation between admission serum creatinine, maximal serum creatinine at day 7 or during hospitalization in ICU, and the pic of creatinine phosphokinase.

## Conclusion

In this multicenter observational study of ICU patients with smoke inhalation, administration of hydroxocobalamin was independently associated with AKI and showed no association with survival. The role of hydroxocobalamin following smoke inhalation needs further consideration and critical appraisal.

## Supplementary information

**Additional file 1 : Table S1.** Coding system for smoke inhalation.

**Additional file 2 : Table S2.** Number of patients from each centers.

**Additional file 3 : Table S3.** Multivariate analysis of factors associated with RRT.

**Additional file 4 : Table S4.** Multivariate analysis of factors associated with major adverse kidney events.

**Additional file 5 : Table S5.** Comparison between admission lactate quartile.

**Additional file 6 : Table S6.** Comparison between severely burn and non-severely burn patients.

## Data Availability

The datasets used and/or analyzed during the current study are available from the corresponding author on reasonable request.

## Abbreviations

AKI: Acute kidney injury
Cis: Confidence intervals
ICU: Intensive care unit
KDIGO: Kidney Disease Improving Global Outcome
MAKE: Major associated kidney events
RRT: Renal replacement therapy
SCreat: Serum creatinine
TBSA: Total body surface area

## References

[1] Lawson-Smith P, Jansen EC, Hyldegaard O (2011). Cyanide intoxication as part of smoke inhalation - a review on diagnosis and treatment from the emergency perspective. Scand J Trauma Resusc Emerg Med.

[2] Petrikovics I, Budai M, Kovacs K, Thompson DE (2015). Past, present and future of cyanide antagonism research: from the early remedies to the current therapies. World J Methodol.

[3] Anseeuw K, Delvau N, Burillo-Putze G, De Iaco F, Geldner G, Holmström P (2013). Cyanide poisoning by fire smoke inhalation: a European expert consensus. Eur J Emerg Med.

[4] Legrand M, Michel T, Daudon M, Benyamina M, Ferry A, Soussi S (2016). Risk of oxalate nephropathy with the use of cyanide antidote hydroxocobalamin in critically ill burn patients. Intensive Care Med.

[5] Legrand M, Mallet V (2017). Intravenous hydroxocobalamin and crystal nephropathy. Nat Rev Nephrol.

[6] Kellum JA, Lameire N, Aspelin P, Barsoum RS, Burdmann EA, Goldstein SL (2012). Work group membership. Kidney Int.

[7] Monafo William W. (1996). Initial Management of Burns. New England Journal of Medicine.

[8] Chou SH, Lin S-D, Chuang H-Y, Cheng Y-J, Kao EL, Huang M-F (2004). Fiber-optic bronchoscopic classification of inhalation injury: prediction of acute lung injury. Surg Endosc.

[9] Bartlett JW, Seaman SR, White IR, Carpenter JR, for the Alzheimer’s Disease Neuroimaging Initiative* (2015). Multiple imputation of covariates by fully conditional specification: accommodating the substantive model. Stat Methods Med Res.

[10] Baud FJ, Borron SW, Mégarbane B, Trout H, Lapostolle F, Vicaut E (2002). Value of lactic acidosis in the assessment of the severity of acute cyanide poisoning. Crit Care Med.

[11] Baud FJ, Haidar MK, Jouffroy R, Raphalen J-H, Lamhaut L, Carli P (2018). Determinants of lactic acidosis in acute cyanide poisonings. Crit Care Med.

[12] Kadri SS, Miller AC, Hohmann S, Bonne S, Nielsen C, Wells C (2016). Risk factors for in-hospital mortality in smoke inhalation-associated acute lung injury: data from 68 United States hospitals. Chest..

[13] Borron SW, Baud FJ, Barriot P, Imbert M, Bismuth C (2007). Prospective study of hydroxocobalamin for acute cyanide poisoning in smoke inhalation. Ann Emerg Med.

[14] Fortin J-L, Giocanti J-P, Ruttimann M, Kowalski J-J (2006). Prehospital administration of hydroxocobalamin for smoke inhalation-associated cyanide poisoning: 8 years of experience in the Paris fire brigade. Clin Toxicol.

[15] Nguyen Lyly, Afshari Ashkan, Kahn Steven A., McGrane Stuart, Summitt Blair (2017). Utility and outcomes of hydroxocobalamin use in smoke inhalation patients. Burns.

[16] EMD Serono, A Division of EMD Inc., Canada. CYANOKIT® Product Monograph. 2014. Available from: http://webprod.hc-sc.gc.ca/dpd-bdpp/index-eng.jsp. Accessed 30 Sept 2014.

[17] Legrand M, Mik EG, Johannes T, Payen D, Ince C (2008). Renal hypoxia and dysoxia after reperfusion of the ischemic kidney. Mol Med Camb Mass.

[18] Legrand M, Almac E, Mik EG, Johannes T, Kandil A, Bezemer R (2009). L-NIL prevents renal microvascular hypoxia and increase of renal oxygen consumption after ischemia-reperfusion in rats. Am J Physiol Renal Physiol.

[19] Soussi S, Dépret F, Benyamina M, Legrand M (2018). Early hemodynamic management of critically ill burn patients. Anesthesiology.

