# Supplementary material for: Association between hydroxocobalamin administration and acute kidney injury after smoke inhalation: a multicenter retrospective study
Source: Crit Care. 2019 Dec 23;23:421. doi: 10.1186/s13054-019-2706-0 (PMC6929494; doi:10.1186/s13054-019-2706-0)
Supplement: Supplementary file 1 — Additional file 1 : Table S1. Coding system for smoke inhalation. [file 13054_2019_2706_MOESM1_ESM.docx]

**Additional file Table 1** Coding system for smoke inhalation

| **Code CIM** | **Definition** |
| --- | --- |
| T599 | Toxic effect of fumes, gases and smoke, unspecified |
| X00·0 | Exposure to smoke, fire and flames |
| X09·0 | Exposure to smoke, fire and flames, unspecified, home |
| X47·0 | Accidental poisoning by carbon monoxide from engine exhaust gases, home |
| X47·08 | Accidental poisoning by carbon monoxide from engine exhaust gases, other specified locations |
| X47·9 | Accidental poisoning by carbon monoxide from engine exhaust gases, unspecified location |
| X67·0 | Auto-intoxication by carbon monoxide from engine exhaust gases |
| X67·9 | Auto-intoxication by unspecified gases and vapours |
| T58 | Toxic effect of carbon monoxide |
