# Supplementary material for: Association between hydroxocobalamin administration and acute kidney injury after smoke inhalation: a multicenter retrospective study
Source: Crit Care. 2019 Dec 23;23:421. doi: 10.1186/s13054-019-2706-0 (PMC6929494; doi:10.1186/s13054-019-2706-0)
Supplement: Supplementary file 2 — Additional file 2 : Table S2. Number of patients from each centers. [file 13054_2019_2706_MOESM2_ESM.docx]

**Additional file Table 2** Number of patients from each centres

| **Centre** | **Number of patients** |
| --- | --- |
| - Percy - Saint-Louis - Garches - Lille - Marseille - Nantes - Metz - Lyon - Bordeaux - Montpellier - Rennes - Le Havre - Orléans - Clermont - Rouen - Caen - Grenoble - Cotentin - HEGP - Limoges - La Rochelle | 219  126  93  63  48  34  33  27  24  12  10  9  8  7  6  5  5  4  3  2  1 |
