# Supplementary material for: Association between hydroxocobalamin administration and acute kidney injury after smoke inhalation: a multicenter retrospective study
Source: Crit Care. 2019 Dec 23;23:421. doi: 10.1186/s13054-019-2706-0 (PMC6929494; doi:10.1186/s13054-019-2706-0)
Supplement: Supplementary file 3 — Additional file 3 : Table S3. Multivariate analysis of factors associated with RRT. [file 13054_2019_2706_MOESM3_ESM.docx]

**Additional file Table 3** Multivariate analysis of factors associated with RRT

| **RRT** | | | | |
| --- | --- | --- | --- | --- |
| **Variable** | **Adjusted Odds ratio** | **LCI** | **UCI** | **p** |
| Hydroxocobalamin | 1.762 | 1.138 | 2.728 | 0.011 |
| Age | 1.003 | 0.990 | 1.016 | 0.663 |
| Peripheral Arterial Obstructive Disease | 0.565 | 0.185 | 1.727 | 0.316 |
| Diabetes mellitus | 1.228 | 0.590 | 2.559 | 0.582 |
| Chronic hypertension | 2.081 | 1.197 | 3.616 | 0.009 |
| Pre-hospital cardiac arrest | 0.924 | 0.057 | 15.084 | 0.956 |
| Severe burn | 2.596 | 1.579 | 4.270 | <0.001 |
| SOFA score at admission without kidney item | 1.058 | 0.937 | 1.195 | 0.359 |
| CKD | 2.690 | 0.342 | 21.176 | 0.347 |
| Catecholamine on admission | 1.188 | 0.609 | 2.315 | 0.613 |
| Pre-hospital GCS | 1.053 | 0.985 | 1.125 | 0.13 |
| Lactate on admission | 1.136 | 1.060 | 1.217 | <0.001 |
| Maximum CPK | 1.099 | 0.930 | 1.298 | 0.26 |
| Aminoglycoside | 2.440 | 1.553 | 3.835 | <0.001 |
| Contrast agent | 0.915 | 0.489 | 1.713 | 0.782 |
| Glycopeptide | 2.835 | 1.299 | 6.188 | 0.009 |
| SAPS2 | 1.022 | 1.007 | 1.038 | 0.006 |

LCI: lower confidence interval, UCI: upper confidence interval, p: p value, RRT: renal replacement therapy, SOFA score: Sequential organ failure assessment score, GCS: Glasgow coma scale, CKD: chronic kidney disease, CPK: creatinine phosphokinase, SAPS2: simplified acute physiology score
