# Supplementary material for: Association between hydroxocobalamin administration and acute kidney injury after smoke inhalation: a multicenter retrospective study
Source: Crit Care. 2019 Dec 23;23:421. doi: 10.1186/s13054-019-2706-0 (PMC6929494; doi:10.1186/s13054-019-2706-0)
Supplement: Supplementary file 4 — Additional file 4 : Table S4. Multivariate analysis of factors associated with major adverse kidney events. [file 13054_2019_2706_MOESM4_ESM.docx]

**Additional file Table 4** Multivariate analysis of factors associated with major adverse kidney events

| **MAKE** | | | | |
| --- | --- | --- | --- | --- |
| **Variable** | **Adjusted Odds ratio** | **LCI** | **UCI** | **p** |
| Hydroxocobalamin | 0.784 | 0.456 | 1.349 | 0.379 |
| Age | 1.043 | 1.026 | 1.059 | <0.001 |
| Peripheral Arterial Obstructive Disease | 3.212 | 0.79 | 13.06 | 0.103 |
| Diabetes mellitus | 1.131 | 0.491 | 2.605 | 0.773 |
| Chronic hypertension | 1.127 | 0.58 | 2.189 | 0.996 |
| CKD | 1.006 | 0.078 | 12.95 | 0.996 |
| SOFA without kidney item | 1.227 | 1.051 | 1.433 | 0.01 |
| Maximum CPK | 1.523 | 1.232 | 1.882 | <0.001 |
| Lactate on admission | 1.141 | 1.039 | 1.252 | 0.006 |
| Pre hospital cardiac arrest  SAPS2 | 0.594  1.027 | 0.004  1.011 | 88.061  1.044 | 0.838  0.001 |
| Glycopeptide during hospitalization | 1.219 | 0.454 | 3.275 | 0.694 |
| Iodized contrast agent | 0.882 | 0.43 | 1.809 | 0.732 |
| Aminoglycoside during hospitalization | 1.356 | 0.781 | 2.353 | 0.279 |
| Pre hospital GCS | 1.084 | 0.998 | 1.176 | 0.055 |
| Severe burn | 2.587 | 1.412 | 4.74 | 0.002 |
| Catecholamine on admission | 1.053 | 0.445 | 2.493 | 0.906 |
| Center | 1.047 | 0.996 | 1.1 | 0.073 |
| Interaction between pre hospital cardiac arrest and hydroxocobalamin | 10.947 | 0.066 | 1813.457 | 0.358 |
|  |  |  |  |  |

MAKE: major associated kidney events, LCI: lower confidence interval, UCI: upper confidence interval, p: p value, CKD: chronic kidney disease; SOFA score: Sequential organ failure assessment score; SAPS2: simplified acute physiology score 2; CPK: creatinine phosphokinase, GCS: Glasgow coma scale
