# Supplementary material for: Association between hydroxocobalamin administration and acute kidney injury after smoke inhalation: a multicenter retrospective study
Source: Crit Care. 2019 Dec 23;23:421. doi: 10.1186/s13054-019-2706-0 (PMC6929494; doi:10.1186/s13054-019-2706-0)
Supplement: Supplementary file 5 — Additional file 5 : Table S5. Comparison between admission lactate quartile. [file 13054_2019_2706_MOESM5_ESM.docx]

**Additional file Table 5** Comparison between admission lactate quartile

| **Characteristics** | **Quartile 1**  **N=176** | **Quartile 2**  **N=168** | **Quartile 3**  **N=170** | **Quartile 4**  **N=167** | **P** |
| --- | --- | --- | --- | --- | --- |
| **At admission**   - Age in years - Sex female n (%) - BMI in Kg/m² - Prehospital cardiac arrest (%) - Prehospital GSC /15 | 46 (32-63)  66 (37.5)  25 (22-28)  1 (0.5)  15 (13-15) | 49 (35-61)  55 (32.7)  24 (22-28)  5 (3)  15 (13-15) | 53 (39-63)  63 (37.1)  24 (22-28)  6 (3.5)  14 (7-15) | 50 (36-63)  66 (39.5)  26 (22-29)  32 (19.2)  12 (3-15) | 0.098  0.6226  0.1927  <0.0001  <0.0001 |
| **Comorbidities:**   - CKD n (%) - CHT n (%) - Diabetes mellitus n (%) - Peripheral artery disease n (%) - CHF n (%) | 0 (0)  34 (19.3)  10 (5.7)  6 (3.4)  7 (3.8) | 2 (1.2)  31 (18.5)  10 (6)  6 (3.6)  10 (6) | 1 (0.6)  35 (20.6)  15 (8.8)  5 (2.9)  6 (3.5) | 2 (1.2)  30 (18)  17 (10)  3 (1.8)  8 (4.8) | 0.5081  0.9331  0.3207  0.7682  0.7237 |
| **Burn characteristic:**   - Burn n (%) - TBSA % - Deep burn TBSA %   SOFA at admission  MAP in mmHg  Vasopressors n (%)  Hydroxocobalamin n (%)  HbCO % | 122 (69.3)  12 (0-22)  1 (0-11)  2 (0-4)  92 (78-105)  25 (14.2)  64 (36.4)  3 (1.6-7) | 130 (77.4)  20 (3-41)  8 (0-25)  3 (1-6)  82 (72-100)  48 (28.6)  91 (54.2)  3.2 (1.7-8.6) | 140 (82.4)  35 (8-60)  20 (0-45)  4 (2-7)  87 (73-102)  53 (31.2)  102 (60)  3.5 (2.2-9.7) | 133 (79.6)  40 (5-61)  20 (0-52)  7 (4-10)  78 (63-96)  92 (55.1)  114 (68.3)  5.5 (2.4-13) | 0.0255  <0.0001  <0.0001  <0.0001  <0.0001  <0.0001  <0.0001  0.0134 |
| **Biological data**   - Plasma lactate in mmol/L - Serum creatinine at admission in µmol/L - Maximal serum creatinine in µmol/L | 1.1 (1-1.4)  70 (56-84)  80 (66-107) | 2.5 (2.1-2.8)  73 (56-93)  97 (71-150) | 4 (3.5-4.5)  76 (59-101)  106 (76-187) | 7.5 (6.1-9.8)  100 (72-123)  132 (100-221) | <0.0001  <0.0001  <0.0001 |
| **Inhalation fibroscopic status n (%)**   - Grade 0 n - Grade 1 n - Grade 2 n - Grade 3 n | 0 (0)  39 (22.2)  22 (12.5)  8 (4.5) | 0 (0)  29 (17.3)  26 (15.5)  20 (11.9) | 1 (0.6)  24 (14.1)  31 (18.2)  16 (9.4) | 0 (0)  22 (13.2)  27 (16.2)  25 (15) | 0.3928  0.1067  0.5266  0.0117 |
| **During ICU hospitalisation**   - In-ICU mortality n (%) - AKI in the first week n (%) - Stage of AKI   - Stage 1 n (%)   - Stage 2 n (%)   - Stage 3 n (%)   - Severe AKI n (%) - RRT at day 7 n (%) - RRT in ICU n (%) - MAKE n (%) - Shock in ICU n (%) - Length of stay in ICU - SAPS2 | 18 (10.2)  36 (20.5)  24 (13.6)  3 (1.7)  9 (5.1)  12 (6.8)  7 (3.8)  18 (10.2)  27 (15.3)  64 (34.7)  19 (3-37)  30 (19-44) | 41 (24.4)  63 (37.5)  20 (11.9)  12 (7.1)  31 (18.5)  43 (25.6)  27 (16.1)  40 (23.8)  59 (35.1)  88 (52.4)  21 (4-51)  36 (25-54) | 68 (40)  82 (48.2)  24 (14.1)  10 (5.9)  48 (28.2)  58 (34.1)  47 (27.6)  58 (34.1)  92 (54.1)  117 (68.8)  20 (3-66)  50 (37-66) | 104 (62.3)  93 (54.7)  28 (16.8)  10 (6)  55 (32.9)  65 (38.9)  51 (30.5)  62 (37.1)  117 (70.1)  121 (72.5)  7 (1-40)  56 (44-72) | <0.0001  <0.0001  0.6413  0.1077  <0.0001  <0.0001  <0.0001  <0.0001  <0.0001  <0.0001  0.0015  <0.0001 |
| **Nephrotoxic in ICU**   - Aminoglycoside during hospitalization - Glycopeptide during hospitalization - Contrast agent | 44 (0.25)  15 (8.5)  12 (6.8) | 49 (29.2)  5 (3)  13 (7.7) | 52 (30.6)  13 (7.7)  20 (11.8) | 34 (20.4)  8 (4.8)  28 (16.8) | 0.1374  0.1145  0.012 |

All data are expressed as median ± 25-75 inter quartile or percentage (%)

BMI: body mass index, GCS: Glasgow coma scale, CKD: chronic kidney disease, CHT: chronic hypertension, CHF: chronic heart failure, TBSA: total body surface area, SOFA: Sequential organ failure assessment, MAP: mean arterial pressure, HbCO: carboxy haemoglobin, ICU: intensive care unit, AKI: acute kidney injury, RRT: renal replacement therapy, MAKE: major associated kidney events, SAPS2: simplified acute physiology score 2
