# Supplementary material for: Association between hydroxocobalamin administration and acute kidney injury after smoke inhalation: a multicenter retrospective study
Source: Crit Care. 2019 Dec 23;23:421. doi: 10.1186/s13054-019-2706-0 (PMC6929494; doi:10.1186/s13054-019-2706-0)
Supplement: Supplementary file 6 — Additional file 6 : Table S6. Comparison between severely burn and non-severely burn patients. [file 13054_2019_2706_MOESM6_ESM.docx]

**Additional file Table 6** Comparison between severely burn and non-severely burn patients

| **Characteristics** | **All patients**  **N=739** | **Non severely burn**  **N=334** | **Severely burn**  **N=405** | **P** |
| --- | --- | --- | --- | --- |
| **At admission**   - Age in years - Sex female n (%) - BMI in Kg/m² - Prehospital cardiac arrest (%) - Prehospital GSC /15 | 50 (36-63)  271 (36.7)  25 (22-28)  46 (6.2)  15 (9-15) | 49 (34-63)  128 (38.3)  24 (22-27)  31 (9.3)  14 (7-15) | 51 (37-62)  143 (35.3)  25 (22-28)  15 (3.7)  15 (12-15) | 0.9678  0.4415  0.0125  0.003  0.0155 |
| **Comorbidities:**   - CKD n (%) - CHT n (%) - Diabetes mellitus n (%) - Peripheral artery disease n (%) - CHF n (%) | 6 (0.8)  141 (19.1)  54 (7.3)  22 (3)  33 (4.5) | 3 (0.9)  69 (20.7)  24 (7.2)  8 (2.4)  15 (4.5) | 3 (0.7)  72 (17.8)  30 (7.4)  14 (3.5)  18 (4.4) | 1  0.3692  1  0.5302  0.621 |
| **Burn characteristic:**   - Burn n (%) - TBSA % - Deep burn TBSA %   SOFA at admission  MAP in mmHg  Vasopressors n (%)  Hydroxocobalamin n (%)  HbCO % | 577 (78.1)  20 (3-47)  9 (0-30)  4 (1-7)  86 (72-101)  226 (30.6)  386 (52.2)  3.6 (1.9-9.7) | 172 (51.5)  1 (0-10)  0 (0-1)  2 (0-6)  92 (76-105)  70 (21)  198 (59.3)  9 (4-18) | 405 (100)  42 (27-62)  30 (14-50)  5 (2-8)  83 (68-98)  156 (38.5)  188 (46.4)  2 (2-4) | <0.0001  <0.0001  <0.0001  <0.0001  <0.0001  <0.0001  0.0007  <0.0001 |
| **Biological data**   - Plasma lactate in mmol/L - Serum creatinine at admission in µmol/L - Maximal serum creatinine in µmol/L | 3.0 (1.8-5.2)  76 (59-101)  100 (73-162) | 2.5 (1.3-4.2)  74 (58-97)  84 (67-119) | 3.5 (2.3-5.7)  79 (60-105)  118 (82-202) | 0.0198  0.6081  <0.0001 |
| **Inhalation fibroscopic status n (%)**   - Grade 0 n - Grade 1 n - Grade 2 n - Grade 3 n | 305 (41.3)  1 (0.1)  121 (16.4)  110 (14.9)  73 (9.9) | 66 (19.8)  1 (0.3)  34 (10.2)  24 (7.2)  7 (2.1) | 239 (59)  0 (0)  87 (21.5)  86 (21.2)  66 (16.3) | <0.0001  0.452  <0.0001  <0.0001  <0.0001 |
| **During ICU hospitalisation**   - In-ICU mortality n (%) - AKI in the first week n (%) - Stage of AKI   - Stage 1 n (%)   - Stage 2 n (%)   - Stage 3 n (%)   - Severe AKI n (%) - RRT at day 7 n (%) - RRT in ICU n (%) - MAKE n (%) - Shock in ICU n (%) - Length of stay in ICU - SAPS2 | 243 (32.9)  288 (39)  102 (13.8)  39 (5.3)  147 (19.9)  186 (25.2)  136 (18.8)  183 (24.8)  313 (42.4)  402 (54.4)  15 (3-44)  42 (27-60) | 50 (15)  66 (19.8)  27 (8.1)  13 (3.9)  26 (7.8)  39 (11.7)  24 (7.2)  31 (9.3)  73 (21.9)  92 (27.5)  6 (2-21)  29 (19-47) | 193 (47.7)  222 (54.8)  75 (18.5)  26 (6.4)  121 (29.9)  147 (36.3)  112 (27.7)  152 (37.5)  240 (59.3)  310 (76.5)  34 (6-67)  49 (35-65) | <0.0001  <0.0001  <0.0001  <0.0001  <0.0001  <0.0001  <0.0001  <0.0001  <0.0001  <0.0001  <0.0001  <0.0001 |
| **Nephrotoxic in ICU**   - Aminoglycoside during hospitalization - Glycopeptide during hospitalization - Contrast agent | 188 (25.4)  41 (5.5)  74 (10) | 36 (10.8)  8 (2.4)  32 (9.6) | 152 (37.5)  33 (8.2)  42 (10.4) | <0.0001  <0.0001  0.816 |

All data are expressed as median ± 25-75 inter quartile or percentage (%)

BMI: body mass index, GCS: Glasgow coma scale, CKD: chronic kidney disease, CHT: chronic hypertension, CHF: chronic heart failure, TBSA: total body surface area, SOFA: Sequential organ failure assessment, MAP: mean arterial pressure, HbCO: carboxy haemoglobin, ICU: intensive care unit, AKI: acute kidney injury, RRT: renal replacement therapy, MAKE: major associated kidney events, SAPS2: simplified acute physiology score 2
